# Fish Distribution and Habitat Complexity on Banks of the Strait of Sicily (Central Mediterranean Sea) from Remotely-Operated Vehicle (ROV) Explorations

**DOI:** 10.1371/journal.pone.0167809

**Published:** 2016-12-09

**Authors:** Pierpaolo Consoli, Valentina Esposito, Pietro Battaglia, Chiara Altobelli, Patrizia Perzia, Teresa Romeo, Simonepietro Canese, Franco Andaloro

**Affiliations:** 1 Laboratorio di ittiologia ed ecologia marina, Istituto Superiore per la Protezione e la Ricerca ambientale (ISPRA), Milazzo, Italy; 2 IV Dipartimento Uso Sostenibile delle Risorse, STS Palermo, Istituto Superiore per la Protezione e la Ricerca Ambientale (ISPRA), Palermo, Italy; 3 III Dipartimento Tutela degli Habitat e della Biodiversità Marina. Istituto Superiore per la Protezione e la Ricerca ambientale (ISPRA) Roma, Italy; Universita degli Studi di Genova, ITALY

## Abstract

The Strait of Sicily was recognized internationally as an “Ecologically or Biologically Significant Area” by the Contracting Parties of the Convention on Biological Diversity in 2014. However, basic aspects of its fish diversity are still unknown and most of the information comes from traditional trawl surveys. This paper provides the first detailed description, using a Remotely Operated Vehicle (ROV), of the composition and depth distribution of the demersal fish assemblages found on banks of the Strait of Sicily and the related habitat complexity from 35 to 240 m depth. A total of 24 families and 52 fish species were recorded and depth was consistently associated with a significant proportion of the variation of the fish assemblage. The highest species richness was observed at the shallowest depth layer (0–50 m) and significantly decreased, remaining almost constant, in deeper layers. Similarly the highest abundance was recorded at 0–50 m, where *C*. *julis* represented the most abundant species, and decreased progressively throughout the whole depth gradient. Although the factor habitat complexity explained only a small proportion of the fish assemblage variation, significant differences among different degrees of habitat complexity were observed, together with a general positive trend for species richness and abundance with increasing habitat complexity. The ROV also allowed us to observe some rare or poorly known fish species such as *Scorpaenodes arenai*, *Hyporthodus haifensis*, *Myliobatis aquila*, *Gadella maraldi*, *Epinephelus caninus* and *Lappanella fasciata*. These findings show that banks serve as reservoirs for fish abundance and biodiversity and that immediate environmental conservation and management actions represent a priority not only for Italy but also for other countries which share the same area.

## Introduction

The Strait of Sicily is located in the central Mediterranean Sea and represents the main link between the Western and Eastern Mediterranean basins. It has a minimum width of about 150 km (between Cape Bon and Mazara del Vallo), a length of about 600 km, and a mean sill of about 400 m depth [1]. It has a highly irregular bottom bathymetry, characterized in the southwest by the wide Tunisian continental shelf and in the northeast by the Sicilian shelf. These two shelves are separated by deep water areas from which arises the volcanic island of Pantelleria [2].

The bottom of the Strait of Sicily is scattered by several submarine elevations (topographically indicated as banks) made up of sedimentary or volcanic rocks [3], that can reach up to 1000 m of height. This complex topography influences the circulation scheme of the Strait characterized by filaments, meanders and eddies, that along the shelf edge of the banks can produce upwelling, locally increasing the biological productivity [4,5] and making this area an important hotspot of biodiversity within the Mediterranean [6,7]. Furthermore, several authors highlighted the presence of important nursery and spawning areas for many fishery resources [7,8,9,10,11,12], in the Strait, mainly where banks are present. These biological features are representative of the sensitivity of this area. The protection of essential fish habitats is one of the most important issues for fishery management in the Strait of Sicily [8,13], given that the human impacts (e.g., fishery, oil exploration and extraction, maritime traffic), diffusion of non-indigenous species and climate change effects are threatening this environment in recent times [14,15]. Recently, during the 40^th^ meeting of the General Fisheries Commission for the Mediterranean Sea (30 May 2016–03 June 2016), a multiannual management plan for the fisheries exploiting European hake and deep-water rose shrimp in the Strait of Sicily was adopted.

Moreover, the whole Strait of Sicily was recognized at international level as an Ecologically or Biologically Significant Area (EBSA) by the Contracting Parties of the Convention on Biological Diversity (CBD) in 2014 (COP12, October, 2014, Pyeongchang, Republic of Korea). In addition, in 2015 during the second RAC/SPA (Regional Activity Centre for Specially Protected Areas), experts started the review of the existing literature on the Strait of Sicily [6,9,10,11,12,16,17,18,19,20,21] to assess the possibility of creating one or more Specifically Protected Areas of Mediterranean Importance (SPAMIs) including these banks. Overall, these environments are poorly investigated owing to difficulties in carrying out scientific surveys and investigations in areas characterized by a rough topography, offshore location, and a strong hydrodynamic regime [22]. Nevertheless, in the last few decades, the employment of increasingly sophisticated remotely operated vehicles (ROV) has allowed to intensify the exploration of banks, including those in the Mediterranean Sea [20,21,22,23,24].

With regards to the banks within the Strait of Sicily, data on the fish fauna is totally lacking and the only information comes from fishery-independent surveys of GRUND (Gruppo Nazionale Demersali) and MEDITS (Mediteranean International Trawl Survey) projects, carried out in neighboring areas on mobile bottoms suitable to trawl fishery [7,9,19], excluding rocky areas with higher complexity (such as banks) and utilizing invasive methods.

The focus of this research was to examine the demersal fish assemblage associated with banks in the Straits of Sicily using a Remotely-Operated Vehicle (ROV), a non invasive technique of exploration, between 35 and 240 m depth. The specific aims were to: 1) describe fish communities associated with banks; 2) quantify patterns of fish assemblage across different depth layers and degrees of habitat complexity; and 3) determine which of these two factors is most important in structuring fish assemblage. The obtained results will contribute to an integrated understanding of the ecology of these banks, in light of their possible inclusion as protected areas.

## Materials and Methods

### Ethics statement

All the data collected in the present study have been gathered by using a non destructive and non invasive Remotely operated Vehicle (ROV). No fish was collected, injured or manipulated. The research, from a formal point of view, has been funded and committed by Sicilian Region and was aimed to assess the marine biodiversity of the Strait of Sicily.

Before sampling, specific authorizations have been granted by the navy officer Mr. Giuseppe Salemme of the Sicilian Navy Headquarter (Marisicilia). This study did not involve endangered or protected species.

### Study areas

In this paper, the term ‘bank’ is used to indicate generically submarine reliefs, such as shallows, ridges, knolls and pinnacles, including the definition of seamounts, submarine elevations of volcanic origin defined by Staudigel et al. [25] as: “any geographically isolated topographic feature on the seafloor taller than 100 m, including ones whose summit regions may temporarily emerge above sea level, but not including features that are located on continental shelves or that are part of other major landmasses”.

**Fig 1:**
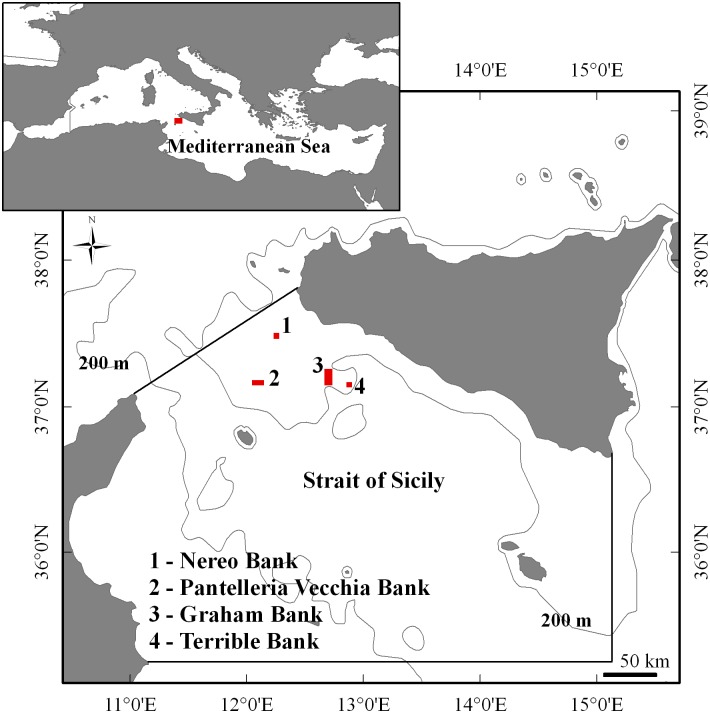
Study area. Investigated banks of the Strait of Sicily following the official delimitation of the Italian Hydrographic Institute of the Navy. (Base map from Natural Earth).

The data on fish communities of the banks of the Strait of Sicily were collected during a research cruise carried out on-board the R/V Astrea of ISPRA, during June-July 2014, in the following areas: Nereo Bank, Pantelleria Vecchia Bank, Graham Bank, Terrible Bank. In Fig 1 the investigated banks in the Strait of Sicily, as delimited by the Italian Navy Hydrographic Institute, are shown.

The Nereo Bank, at about 15 miles from the south-west coast of Sicily, is one of the numerous shallows spotting the Adventure plateau: the wide and flat continental platform of carbonatic origin in the north-western sector of the Strait of Sicily [26,27]. The Nereo Bank ranges from 30 to 60 m depth and the northern main ridge covers an area of approximately 1.4 km^2^. The Pantelleria Vecchia Bank, made of sandstones, is located at 18 miles from Pantelleria Island. It is composed of two main shoals, varying from 16 to 24 meters depth; the surrounding areas are located at depths ranging from 46 to 60 meters [28]. The Graham Bank, along with Terrible Bank and Nerita Bank, is part of a large volcanic relief named Empedocle Seamount. The Graham Bank is composed by numerous volcanic edifices, such as the ephemeral Ferdinandea Island, located about 16 nautical miles from south-west coast of Sicily. The volcanic cones range from 9 meters and 250 meters [29,30]. The Terrible Bank is located about 20 miles from the south-west coast of Sicily and 40 miles from Pantelleria; its summit is around 20 meters depth.

### Field sampling methods

Data on fish fauna and habitats were collected through a Remotely-Operated Vehicle (ROV ‘‘PolluxIII”) equipped with a digital camera (Canon EOS 5D, 20 megapixel), Two strobe (Canon), a high definition video camera (Sony HDR-HC7), and 3 jaw grabbers.

The ROV also hosted a depth sensor, a compass, and three parallel laser beams providing a 10-cm scale for the measurements and it was equipped with an underwater acoustic tracking position system (Tracklink 1500 MA, Link Quest Inc.) providing geographic position of the ROV along the seabed.

Transects were of variable lengths; some were short, interrupted by frequent sampling, and some were longer, covering wide ranges of habitats. The video camera recorded continuously onto hard drives throughout the dives, and digital still images were taken frequently to augment the video. ROVs were used to visually assess fish occurrence, distribution and habitat complexity on the banks. A total of 13 dives were executed, providing over almost 11 h of bottom imagery. Fish abundance was estimated by counting single specimens up to a maximum of 10 individuals, and using abundance-classes (11–30, 31–50, 51–100, 101–200, 201–500, 500) for schools. This recording system leads to a similar degree of error over a wide range of abundances, ensuring homogeneity of variance after log-transformation of the data [31].

### Video and data analysis

Direct observation methods are preferred for documenting fauna in complex habitats [32–34]; thus, ROV video recordings were the main data used to document the fish communities and associated habitat complexity in the study area. ROV dive tracks were initially processed to conservatively remove erroneous tracking data (location points). Dive videos were analyzed multiple times for habitat classifications and to document necto-benthic fishes to the lowest possible taxon. Unusable videos (out of focus, too far off bottom, video malfunction, sediment clouds) were removed from the dataset [34].

**Fig 2:**
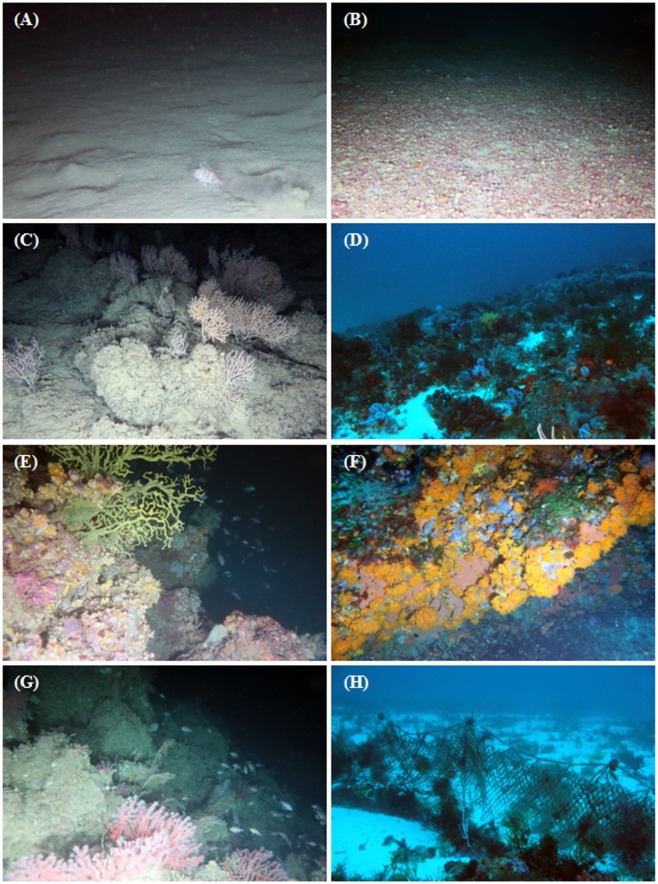
In situ photographs of benthic habitats in banks of the Strait of Sicily. (A) *Helicolenus dactylopterus* on soft sediment, low relief; (B) rhodolith beds, low relief; (C) colonies of *Eunicella cavolinii* on medium relief bedrock; (D) Macrofauna assemblage forming a medium relief habitat; (E) *Savalia savaglia* on high relief bedrock with a school of *Anthias anthias* in the background; (F) high relief coralligenous habitat; (G) school of *A*. *anthias* swimming close to fan-shaped *Corallium rubrum* on a high complexity habitat; (H) Ghost net on soft sediment.

From 13 dives a total of 181 video segments were extracted as sample units. First of all, data were divided into four depth layers (0–50, 51–100, 101–150, 151–200 m) and within each layer, video segments were designated when the habitat complexity changed on the basis of three levels (low, medium and high; Table 1; Fig 2) [33,34]. Following this procedure, a video segment (sample unit) was characterized by univocal category of depth layer and habitat complexity. Number of individuals for each species and total number of species (species richness) were calculated for each video segment. Sample units with no species observed were removed from the dataset. Since transect times were variable, species' abundances were standardized per sample unit by dividing the number of individuals of each species by the total number of fishes per sample. Standardized abundances were fourth root transformed to down weight the common species relative to the rare species. Similarities among samples were calculated using a Bray–Curtis similarity coefficient [35].

**Table 1:** Description of three levels of the factor Habitat complexity.

Levels	Characteristics
**Low**	Soft sediment with/out current ripples, maërl beds, rubbles, gravel; flat hard substrata; low profile.
**Medium**	Soft substrata with scattered boulders and/or pebbles and rocky outcrops. Hard substrata with algae and *Posidonia* meadows, benthic invertebrates, pebbles, gravel; medium profile, height < 100 cm
**High**	Soft substrata with close boulders and/or rocky outcrops; bedrock with walls, ridges, cavities, caves; steeply sloping; high profile, height > 100 cm

DistLM marginal tests were used to determine the extent to which habitat complexity and depth explained a proportion of the variation in the assemblage structure. Habitats were coded as nominal, binominal categories and grouped as an indicator for this analysis [36]. Then, two different one-way permutational multivariate analyses of variance (PERMANOVA) [36,37,38] were used to test the differences among fish assemblages with regard to the factors “habitat complexity” and “depth layer”. Significant terms were investigated using *a posteriori* pair-wise comparisons with the PERMANOVA t statistic and 999 permutations. Non-metric multidimensional scaling ordination plots (MDS), were also created for factor Depth and Habitat complexity and SIMPER analysis was used to determine which species contributed to the dissimilarities among levels of habitat complexity and depth.

Statistical analyses were accomplished for thirty-four species of fish because species with less than five specimens, species that were observed only once and highly gregarious species were excluded (see S1 Dataset). All multivariate analyses were conducted by PRIMER 6 with the PERMANOVA + add-on program package [39]. Finally, the abundance of each species (see S2 Dataset) was correlated with depth and complexity by correspondence analyses (CA) performed using the software package STATISTICA, version 10 [40]. The preferred depth for thirty fish species was assessed by calculating the weighted average depth:

$$\;\frac{\sum_{\text{i}=1}^{\text{n}}{\text{p}}_{\text{i}}{\text{x}}_{\text{i}}}{\sum_{\text{i}=1}^{\text{n}}{\text{p}}_{\text{i}}}$$

where p_i_ is the number of individual of species i recorded at the depth x_i_ (see S3 Dataset).

According to the available literature, each species was assigned to one of the following trophic guilts: planktivores (Pla), piscivores-benthivores (Pis-ben), benthivores (Ben), and piscivores (Pis).

## Results

Overall 24 families and 52 fish species (1 elasmobranch and 51 bony fishes) were recorded in the study area (Table 2; S4 Dataset). The fish species mainly belonged to Labridae (13), Serranidae (7), Scorpaenidae (5) and Sparidae (5). The most speciose genus was *Symphodus*, with 6 species, followed by *Scorpaena* (4 species), *Epinephelus* and *Labrus* (3 species). Benthivores composed the richest guild (26), followed by piscivores-benthivores (16), planktivores (8) and piscivores (2). The gregarious planktivores species *Anthias anthias* and *Chromis chromis* represented 66% of total individuals, whereas the benthivorous *Coris julis* reached 21% of the total fish abundance, followed by *Diplodus vulgaris* (0.9%) and *Serranus cabrilla* (0.8%).

**Table 2:** Percent relative abundance of fishes observed on banks of the Strait of Sicily within four depth ranges and three habitat complexities.

Taxa		Trophic guild	Depth range				Complexity		
			0–50	51–100	101–150	151–200	low	medium	high
***Actinopterygii***									
Apogonidae									
*Apogon imberbis* (Linnaeus 1758)	*Aimb*	Pla	0.12	0.00	0.00	0.00	0.00	0.00	0.08
Aulopidae									
*Aulopus filamentosus* (Bloch, 1792)	*Afil*	Pis-ben	0.00	0.03	0.21	1.54	0.33	0.08	0.06
Blennidae									
*Parablennius rouxi* (Cocco, 1833)	*Prou*	Ben	0.01	0.00	0.00	0.00	0.00	0.02	0.00
Bothidae									
*Bothidae* und.	*Bund*	Ben	0.00	0.00	0.00	0.51	0.00	0.02	0.00
Callanthidae									
*Callanthias ruber* (Rafinesque, 1810)	*Crub*	Pla	0.00	0.27	20.54	53.85	5.06	6.05	4.87
Caproidae									
*Capros aper* (Linnaeus, 1758)	*Cape*	Pla	0.00	0.00	0.00	1.03	0.33	0.00	0.00
Carangidae									
*Seriola dumerili* (Risso, 1810)	*Sdum*	Pis	0.09	0.00	0.00	0.00	0.00	0.10	0.00
*Trachurus trachurus* (Linnaeus, 1758)	*Ttra*	Pis-Pla	0.00	5.55	0.00	0.00	0.00	0.00	3.32
Centracanthidae									
*Spicara maena* (Linnaeus, 1758)	*Smae*	Pla	0.22	0.00	0.00	0.00	0.00	0.25	0.00
*Spicara smaris* (Linnaeus, 1758)	*Ssma*	Pla	0.38	0.00	0.00	0.00	0.00	0.43	0.00
Centriscidae									
*Macroramphosus scolopax* (Linnaeus, 1758)	*Msco*	Ben	0.00	0.98	0.23	5.13	1.14	0.20	0.59
Congridae									
*Conger conger* (Linnaeus, 1758)	*Ccon*	Pis-ben	0.00	0.05	0.03	0.00	0.00	0.03	0.02
Gobiidae									
*Gobiidae* und.	*Gund*	Ben	0.06	0.02	0.03	0.00	0.33	0.07	0.00
Labridae									
*Coris julis* (Linnaeus, 1758)	*Cjul*	Ben	42.73	10.27	0.26	0.00	65.25	42.81	5.77
*Ctenolabrus rupestris* (Linnaeus, 1758)	*Crup*	Ben	0.01	0.00	0.00	0.00	0.00	0.02	0.00
*Labrus merula* Linnaeus, 1758	*Lmer*	Ben	0.35	0.00	0.00	0.00	0.00	0.36	0.02
*Labrus mixtus* Linnaeus, 1758	*Lmix*	Ben	0.04	0.00	0.00	0.00	0.00	0.00	0.03
*Labrus viridis* Linnaeus, 1758	*Lvir*	Ben	0.03	0.00	0.00	0.00	0.00	0.03	0.00
*Lappanella fasciata* (Cocco, 1833)	*Lfas*	Ben	0.00	0.11	0.29	2.05	0.33	0.05	0.16
*Symphodus doderleini* Jordan, 1890	*Sdod*	Ben	0.01	0.00	0.00	0.00	0.00	0.02	0.00
*Symphodus mediterraneus* (Linnaeus, 1758)	*Smed*	Ben	0.39	0.05	0.00	0.00	0.00	0.41	0.05
*Symphodus melanocercus* (Risso, 1810)	*Smel*	Ben	0.36	0.02	0.00	0.00	0.00	0.39	0.02
*Symphodus ocellatus* (Forsskål, 1775)	*Soce*	Ben	0.39	0.00	0.00	0.00	0.00	0.43	0.01
*Symphodus roissali* (Risso, 1810)	*Sroi*	Ben	0.19	0.00	0.00	0.00	0.00	0.20	0.01
*Symphodus tinca* (Linnaeus, 1758)	*Stin*	Ben	0.48	0.00	0.00	0.00	0.00	0.49	0.03
*Thalassoma pavo* (Linnaeus, 1758)	*Tpav*	Ben	0.01	0.00	0.00	0.00	0.00	0.02	0.00
Moridae									
*Gadella maraldi* (Risso, 1810)	*Gmar*	Ben	0.00	0.00	0.67	1.03	0.33	0.28	0.09
Mullidae									
*Mullus surmuletus* Linnaeus, 1758	*Msur*	Ben	0.16	0.00	0.03	0.00	0.33	0.10	0.04
Muraenidae									
*Muraena helena* Linnaeus, 1758	*Mhel*	Pis-ben	0.00	0.03	0.05	0.00	0.00	0.00	0.04
Phycidae									
*Phycis phycis* (Linnaeus, 1766)	*Pphy*	Pis-ben	0.01	0.08	0.26	0.51	0.33	0.00	0.14
Pomacentridae									
*Chromis chromis* (Linnaeus, 1758)	*CChr*	Pla	22.89	0.00	0.00	0.00	1.63	19.29	3.79
Scorpaenidae									
*Scorpaena elongata* Cadenat, 1943	*Selo*	Pis-ben	0.00	0.00	0.00	0.51	0.00	0.02	0.00
*Scorpaena maderensis* Valenciennes, 1833	*Smad*	Ben	0.03	0.00	0.00	0.00	0.00	0.00	0.02
*Scorpaena notata* (Rafinesque, 1810)	*Snot*	Pis-ben	0.00	0.00	0.00	0.51	0.00	0.02	0.00
*Scorpaena scrofa* Linnaeus, 1758	*Sscr*	Pis-ben	0.00	0.02	0.13	1.03	0.16	0.05	0.04
*Scorpaenodes arenai* Torchio, 1962	*Sare*	Ben	0.00	0.00	0.03	0.00	0.00	0.00	0.01
Sebastidae									
*Helicolenus dactylopterus* (Delaroche, 1809)	*Hdac*	Pis-ben	0.00	0.00	0.05	2.56	0.65	0.02	0.02
Serranidae									
*Anthias anthias* (Linnaeus, 1758)	*Aant*	Pla	24.22	82.05	76.55	28.72	18.76	21.47	79.98
*Epinephelus caninus* (Valenciennes, 1843)	*Ecan*	Pis-ben	0.00	0.00	0.08	0.00	0.00	0.00	0.03
*Epinephelus costae* (Steindachner, 1878)	*Ecos*	Pis-ben	0.04	0.00	0.00	0.00	0.00	0.02	0.02
*Epinephelus marginatus* (Lowe, 1834)	*Emar*	Pis-ben	0.03	0.00	0.00	0.00	0.00	0.03	0.00
*Hyporthodus haifensis* (Ben-Tuvia, 1953)	*Hhai*	Pis-ben	0.00	0.00	0.05	0.00	0.00	0.00	0.02
*Serranus cabrilla* (Linnaeus, 1758)	*Scab*	Pis-ben	1.31	0.44	0.52	0.00	4.57	1.23	0.34
*Serranus scriba* (Linnaeus, 1758)	*Sscr*	Pis-ben	0.07	0.00	0.00	0.00	0.00	0.08	0.00
Sparidae									
*Boops boops* (Linnaeus, 1758)	*Bboo*	Pla	2.67	0.00	0.00	0.00	0.00	3.03	0.00
*Diplodus vulgaris* (Geoffroy Saint-Hilaire, 1817)	*Dvul*	Ben	2.16	0.00	0.00	0.00	0.00	1.79	0.39
*Pagellus bogaraveo* (Brunnich, 1768)	*Pbog*	Pis-ben	0.00	0.00	0.00	0.51	0.16	0.00	0.00
*Sparus aurata* Linnaeus, 1758	*Saur*	Ben	0.00	0.02	0.00	0.00	0.00	0.00	0.01
*Spondyliosoma cantharus* (Linnaeus, 1758)	*Scan*	Ben	0.07	0.00	0.00	0.00	0.00	0.08	0.00
Triglidae									
*Chelidonichthys cuculus* (Linnaeus, 1758)	*Acuc*	Ben	0.01	0.00	0.00	0.00	0.16	0.00	0.00
Zeidae									
*Zeus faber* Linnaeus 1758	*Zfab*	Pis	0.01	0.02	0.00	0.51	0.16	0.02	0.01
***Elasmobranchii***									
Myliobatidae									
*Myliobatis aquila* (Linnaeus, 1758)	*Maqu*	Ben	0.43	0.00	0.00	0.00	0.00	0.02	0.00
**Total relative abundance %**			**40.08**	**36.49**	**22.30**	**1.13**	**3.55**	**35.33**	**61.12**
**Species richness %**			**39.76**	**20.48**	**21.69**	**18.07**	**20.00**	**44.44**	**35.56**
**Species richness**			**33**	**17**	**18**	**15**	**18**	**40**	**32**
Total time (min)			**3.20**	**2.30**	**2.58**	**1.54**	**2.30**	**5.30**	**2.42**
N. of video segments			**92**	**30**	**46**	**13**	**27**	**95**	**59**

Multivariate analysis indicated a strong influence of depth in the assemblage structure: it explained 34% of the variability in the assemblage structure (DistLM marginal test. p = 0.001). Total abuldance was highest at 0–50 m and decreased as depth increased. The majority of biodiversity was observed in the upper layer whereas it remained constant in all the others (Table 2). PERMANOVA analysis showed highly significant differences in the composition of fish assemblage (p = 0.001) for the factor Depth and pair-wise tests revealed that these differences were always significant except for the comparisons between the two deeper layers (101–150 vs 151–200; p = 0.241).

**Fig 3:**
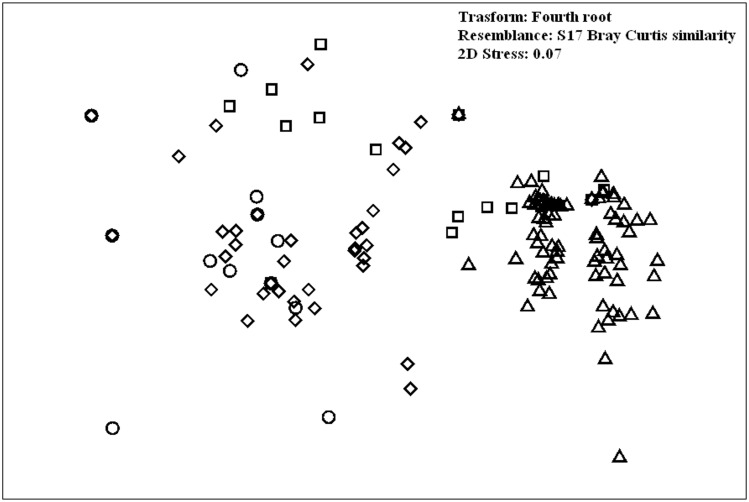
Multidimensional Scaling (MDS) of the composition of fish assemblages among different depth ranges. Triangle = 0–50 m; square = 51–100 m; rhombus = 101–500 m; circle = 151–200 m.

The nMDS biplot (Fig 3), showing the ordination of sampling points by factor depth, evidenced a separation among 0–50 m and all the other depth layers. The sampling points relative to this range were closer to each other because the variability inside this group was very low. SIMPER procedure pinpointed some fish taxa as the major contributors to the differences between depth layers. High densities of *C*. *julis* and *D*. *vulgaris* characterized the censuses carried out at 0–50 m of depth; both species mostly influenced the differences among these layers and all the others. In the second layer (51–100 m) *C*. *julis* was still abundant and together with *S*. *cabrilla* differentiated the fish communities of this layer. Finally, *Callanthias ruber* was very abundant under 100 m depth, and accounted for a large percentage of the dissimilarities between the deeper layers and the shallower ones (Table 3).

**Table 3:** SIMPER of fish taxa contributing most (%) to dissimilarity among the four levels of factor Depth.

**Species**	**Group 0–50 Av. Abund**	**Group 51–100 Av. Abund**	**Contrib %**	**Cum.%**
*Coris julis*	0.88	0.57	28.31	28.31
*Serranus cabrilla*	0.15	0.21	17.14	45.46
*Diplodus vulgaris*	0.11	0	6.46	51.92
**Species**	**Group 0–50 Av. Abund**	**Group 101–150 Av. Abund**	**Contrib %**	**Cum.%**
*Coris julis*	0.88	0.02	33.67	33.67
*Callanthias ruber*	0	0.64	24.89	58.56
*Serranus cabrilla*	0.15	0.16	9.1	67.66
**Species**	**Group 0–50 Av. Abund**	**Group 151–200 Av.Abund**	**Contrib %**	**Cum.%**
*Coris julis*	0.88	0	32.91	32.91
*Callanthias ruber*	0	0.54	19.99	52.9
*Helicolenus dactylopterus*	0	0.16	6.14	59.04
**Species**	**Group 51–100 Av. Abund**	**Group 101–150 Av. Abund**	**Contrib %**	**Cum.%**
*Callanthias ruber*	0.07	0.64	27.44	27.44
*Coris julis*	0.57	0.02	25.45	52.89
*Serranus cabrilla*	0.21	0.16	12.39	65.28
**Species**	**Group 51–100 Av. Abund**	**Group 151–200 Av.Abund**	**Contrib%**	**Cum.%**
*Coris julis*	0.57	0	24.34	24.34
*Callanthias ruber*	0.07	0.54	22.36	46.71
*Serranus cabrilla*	0.21	0	9.04	55.74
**Species**	**Group 101–150 Av. Abund**	**Group 151–200 Av.Abund**	**Contrib%**	**Cum.%**
*Callanthias ruber*	0.64	0.54	26.94	26.94
*Gadella maraldi*	0.11	0.12	11.7	38.64
*Macroramphosus scolopax*	0.07	0.16	10.6	49.24

**Fig 4:**
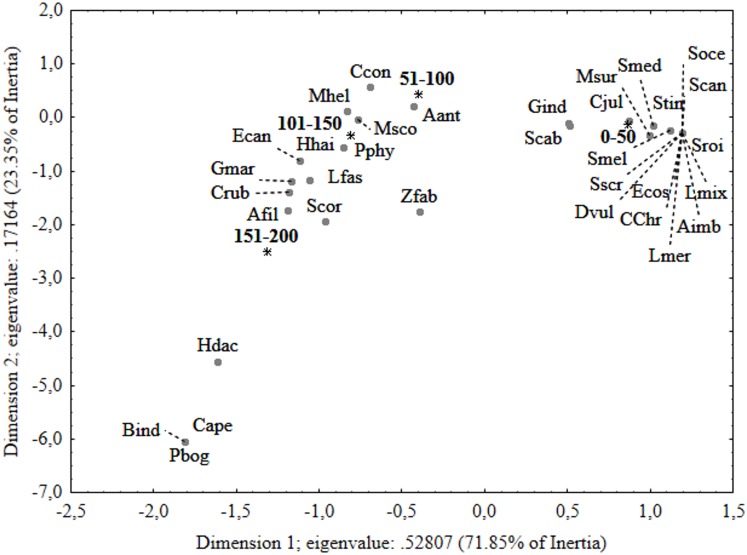
Correspondence analysis (CA) per sampling depth. Ordination diagrams for the first two canonical axes of the correspondence analysis performed on species relative abundance data per sampling depth. Species codes as in Table 2.

The results of PERMANOVA and SIMPER analyses agree with the ordination of fish species in relation to depth strata in the correspondence analysis biplot (Fig 4). The first two axes of the graph explained 95.2% of the total variance. The first dimension highlighted a clear separation between the 0–50 m strata and the other depths: it was due to the high number of species (11) observed exclusively at this lowest depth layer and to the high abundances of *C*. *julis* and *C*. *chromis*. The second dimension showed a separation between 51–100 m (top left in the diagram) and the two deeper strata (101–150 and 151–200 m). *A*. *anthias* was mostly associated with the 51–100 m depth layer, while *C*. *ruber* showed a preference for the two deeper layers. Moreover, two fish species, *Pagellus bogaraveo* and *Capros aper*, located in the bottom left of the diagram, were exclusively observed at 151–200 m depth.

**Fig 5:**
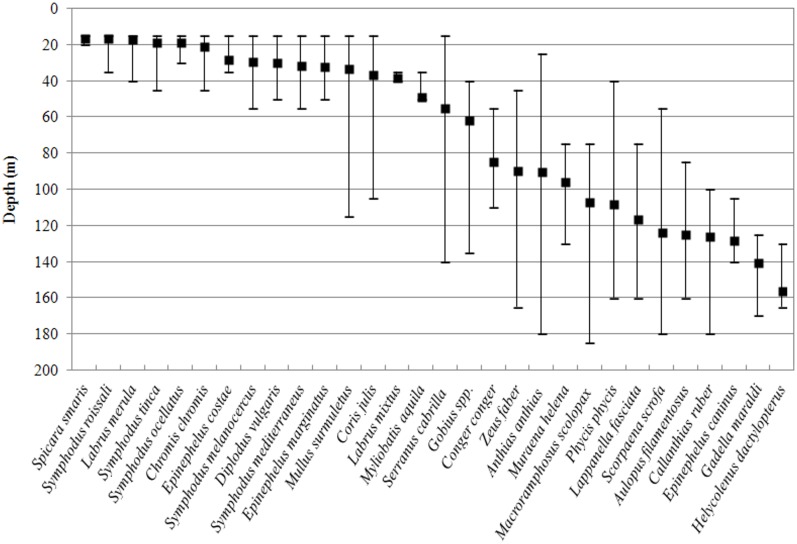
Preferred depth for fish on banks of the Strait of Sicily. Weighted average depth ± max/min depth.

The preferred depth of each fish species, expressed as mean depth of occurrence weighted by abundance, is shown in Fig 5. Overall, depth range is fairly consistent with results from the correspondence analysis (Fig 4): most of the species found at 0–50 m have a smaller distributional range than deeper species. The largest depth ranges were observed for *A*. *anthias* (155 m), *S*. *cabrilla* and *Scorpaena porcus* (125 m), *Phycis phycis* and *Zeus faber* (120 m), *Macroramphosus scolopax* (110 m) and *Mullus surmuletus* (100 m).

**Fig 6:**
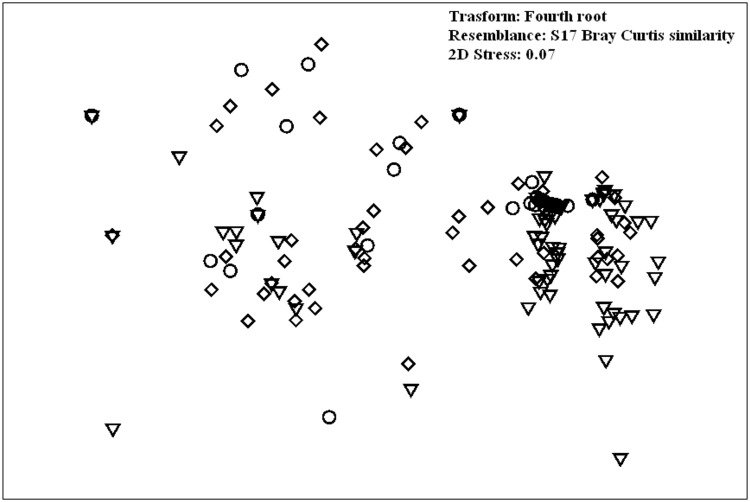
Multidimensional Scaling (MDS) of the composition of fish assemblages among different degrees of habitat complexity. Circle = low complexity; triangle = medium complexity; rhombus = high complexity.

Multivariate analysis of fish assemblage structure among degrees of habitat complexity revealed a progression of assemblages from simple to more complex habitats. However this factor explained only a low proportion of the variation (2.5%, DistLM marginal test, p = 0.005) in assemblage structure. Although groupings are less clear than for depth zonation (Fig 6), there was a significant difference in assemblage across habitat complexity (PERMANOVA, p = 0.001). In this case, pairwise comparisons showed a significant difference of assemblages associated with different levels of spatial complexity.

According to SIMPER analysis (Table 4), *C*. *julis*, *S*. *cabrilla* and *C*. *ruber* cumulatively contributed for more than 48% of the dissimilarity of each comparison between complexity levels. Interestingly, the greatest dissimilarity was observed between assemblages associated with the lowest and the highest spatial complexity. As regards total abundance, a positive trend was also observed with increasing complexity with 61% of the total abundance associated with the highest spatial complexity. At the same time the lowest number of species was observed on low complex habitats.

**Table 4:** SIMPER of fish taxa contributing most (%) to dissimilarity among the three levels of factor Habitat complexity.

**Species**	**Group Low Av.Abund**	**Group Medium Av.Abund**	**Contrib%**	**Cum.%**
*Coris julis*	0.54	0.69	21.57	21.57
*Serranus cabrilla*	0.38	0.24	16.55	38.12
*Callanthias ruber*	0.14	0.16	11.41	49.53
**Species**	**Group Low Av.Abund**	**Group High Av.Abund**	**Contrib%**	**Cum.%**
*Coris julis*	0.54	0.4	20.63	20.63
*Callanthias ruber*	0.14	0.36	16.63	37.26
*Serranus cabrilla*	0.38	0.19	15.3	52.56
**Species**	**Group Medium Av.Abund**	**Group High Av.Abund**	**Contrib%**	**Cum.%**
*Coris julis*	0.69	0.4	20.63	20.63
*Callanthias ruber*	0.16	0.36	16.37	37
*Serranus cabrilla*	0.24	0.19	11.7	48.7

**Fig 7:**
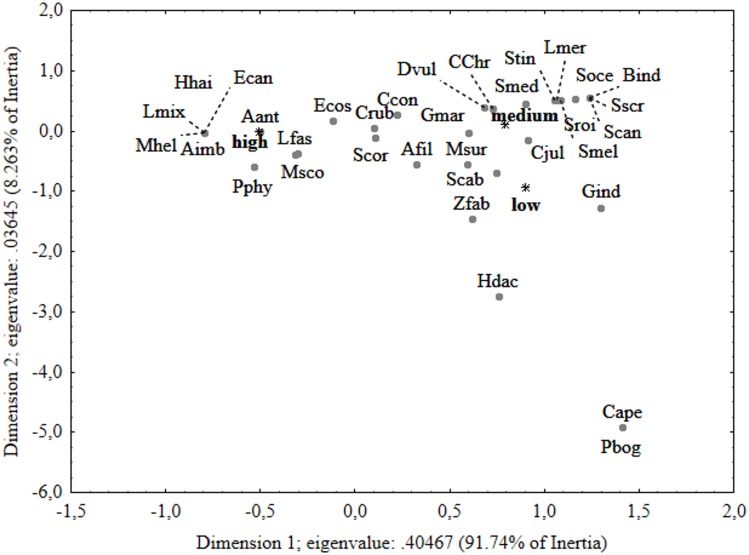
Correspondence analysis (CA) per Habitat complexity. Ordination diagrams for the first two canonical axes of the correspondence analysis performed on species relative abundance data per Habitat complexity. Species codes as in Table 2.

The ordination of fish species in relation to habitat complexity in the correspondence analysis biplot (Fig 7) agrees with results of PERMANOVA and SIMPER analyses. The first two axes of the graph explained 100% of the total variance. The first dimension highlighted a separation between the fish assemblage observed in association to high complexity and the other two, mainly related to the huge abundances of *A*. *anthias* and to four species (*Apogon imberbis*, *Epinephelus caninus*, *Murena helena* and *Hyporthodus haifensis*) exclusively observed in association with this level of complexity. Although fish assemblages related to low and medium complexity levels appeared close to each other in the CA plot, the two species, *C*. *aper* and *P*. *bogaraveo*, located at the bottom right of the diagram, were exclusively observed in low complexity habitats. Moreover, the fish assemblage observed at medium complexity was characterized by high relative abundances of *C*. *julis* and *C*. *chromis*.

### Noteworthy records

**Fig 8:**
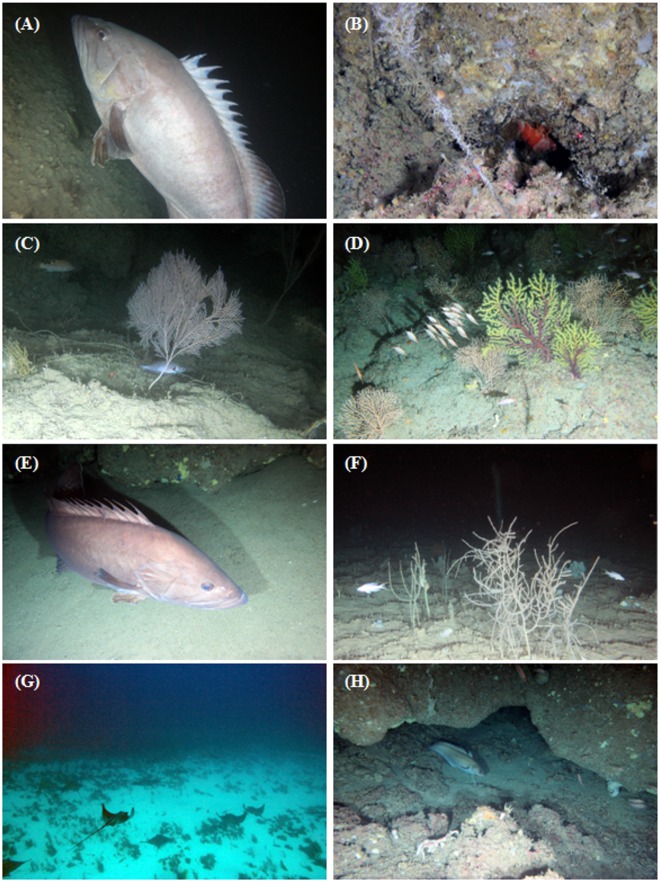
Representative demersal fish species from the banks of the Strait of Sicily. (A) *Epinephelus caninus*; (B) *Scorpaenodes arenai*; (C) *Gadella maraldi*; (D) *Macroramphosus scolopax*; (E) *Hyporthodus haifensis*; (F) *Callanthias ruber*; (G) *Myliobatis aquila*; (H) *Phycis phycis*.

Some rare or poorly known fish species (*Scorpaenodes arenai*, *Gadella maraldi*, *Hyporthodus haifensis*, *Myliobatis aquila*) were observed during this study. Indeed, ROV explorations allowed to observe for the first time *S*. *arenai* (Fig 8B) in its preferential habitat, i.e. small crevices of high complex rocky bottom (further details are given in Battaglia *et al*. [18]). Small caves of hard substrata characterized by medium complexity were inhabited by *G*. *maraldi* (Fig 8C) which was usually observed in depths between 100 and 150 m. Similarly to *S*. *arenai*, also *G*. *maraldi* hides in caves and showed an elusive behavior when the ROV tried to approach them, swimming fast into the crevices. Two large specimens of the thermophile fish *H*. *haifensis* (Fig 8E) were also observed at 150 m on igneous rocky bottom at high complexity level. Moreover, several specimens of *M*. *aquila* (Fig 8G) swimming in groups were encountered during two ROV dives on Nereo and Pantelleria Vecchia Banks, at a depth of 50 and 35 m respectively.

Finally, the ROV explorations enabled identification of several areas of banks of the Strait of Sicily impacted by lost fishing gears (Fig 2H), in particular on Graham Bank and on hard bottoms characterized by high spatial complexity. Lost fishing gears, observed in almost 10% of the video segments, included both lines (mostly longlines) and nets (bottom trawls, set nets) and often entangled soft and hard coral colonies.

## Discussion

The recent technological enhancement of remotely-operated vehicles (ROVs), which takes advantage of high definition video and digital cameras, is improving the biodiversity assessment of fish and benthic invertebrates especially in natural and artificial complex habitats, where it is difficult to use traditional sampling gears [6,32,33,34,41,42] and in deep environments, where diving is logistically limited and the use of submersibles is very expensive. Moreover, direct observation methods allow assessment of behaviors and other attributes, albeit brief, that are otherwise unattainable.

The analysis of video footage, collected on banks of the Straits of Sicily allowed the exceptional opportunity to study, for the first time, the diversity of fish fauna inhabiting the area, implementing non-invasive methods without any impact on fish and benthic assemblage. Therefore, this study adds new and important information regarding those areas which have not been studied in the past, because of the difficulties in collecting data on rough bottom by traditional sampling methods. Indeed, current knowledge has more focused on demersal fish living on soft bottoms of neighboring areas, by means of studies on trawl surveys, aimed to collect data on the status of exploitation of main commercial species [19].

As found in similar studies ([34,43,44] and references therein), depth was the variable that contributed most to structure fish assemblages In agreement with these findings, the present research, supported by multivariate analysis, revealed significant differences among fish assemblages associated with different depth layers. However, the separation among groups in the MDS was not always so well defined, (indicating gradual changes in species composition) probably because many fish species, especially deepest ones, showed broad and overlapping depth ranges. There are several factors that might explain these depth differences between fish species: the most important are diet specialization and interspecific competition [45,46,47].

The highest species richness was observed at the shallowest depth layer (0–50 m) and decreased significantly, remaining almost constant, in the deepest layers. Similarly the highest abundance was recorded at 0–50 m, where *C*. *julis* represented the most abundant species, and decreased progressively throughout the whole depth gradient. Decreasing trends with depth for species richness and abundance have also been reported by several authors outside and inside the Mediterranean Sea [48,49,50].

Although the Habitat complexity explained only a small proportion of the fish assemblage variation, the multivariate analyses showed significant differences among different degrees of habitat complexity. In particular, a general positive trend was observed for species richness and abundance with the increasing habitat complexity. Similar relationships among spatial complexity, fish abundance and species richness have also been worldwide reported ([51,52,53,54] and references therein). The main mechanism invoked to explain these evidences is a reduction of predation pressure due to the increased amount of refuge available for prey species [55,56,57]. Increase in available refuges due to enhanced substrate topography also has been shown to reduce competition for space [55,58] as well as adding to niche dimensionality [59], both of which potentially increase fish abundance and distribution.

The present study allowed to observe, by ROV, some rare or poorly known fish species in their habitat. The occurrence of *S*. *arenai* in the Strait of Sicily (Graham Bank) was recorded for the first time by underwater observation (further details were given by Battaglia *et al*. [18]). This species had been considered endemic from the Strait of Messina until 1995, when some individuals were collected in the Azores waters (Atlantic Ocean) [7], and no other records from Mediterranean waters have been reported out of the Strait of Messina until the contribution of Battaglia *et al*. [18]. According to these authors, the particular habitat and behavior of *S*. *arenai*, consisting in lying upside-down, belly up at the entrance of small crevices looking for potential prey, may be the main reason of the lack of data on this species. ROV investigations allowed to observe two large specimens of *H*. *haifensis* in their habitat and together with other recent records [6,60] of this thermopile grouper support the hypothesis of a northernmost expansion of the species in the Mediterranean Sea. The banks of the Strait of Sicily could represent a recovery area for this species and other groupers observed during this study (*Epinephelus caninus*, *E*. *costae*, *E*. *marginatus*), considering the difficulty to exploit this kind of fishing ground and the distance of these areas from mainland. The exceptional observation of large groups of *M*. *aquila* allows to include the Nereo and Pantelleria Vecchia Banks as a potential area of aggregation of this species. At the light of the fact that the Mediterranean population of *M*. *aquila* is assessed as Vulnerable in the IUCN red list [61], these banks should be considered as places where monitoring of this rare species is required.

The data emerging from this research are essential for the beginning of a long term marine management process, geared towards both a further characterization of the living communities associated with the banks of the Strait of Sicily, and an evidence-based proposal to limit certain fishing activities within the surveyed areas. Despite the presence of large carnivorous fishes such as groupers, indications of fishing impacts are witnessed by the several lost fishing gears (lines, set net, trawl net) and by the habitat damages, resulting from these activities observed on the bottom (pers. obs.). On the other hand, some poorly known species (*S*. *arenai*, *H*. *haifensis*, *M*. *aquila*, *G*. *maraldi*, *E*. *caninus*, *L*. *fasciata*) and species of great concern to fisheries (mostly groupers of different species; [11,17]), were only seen on complex habitats and mostly at deeper layers (especially on volcanic banks), where, generally it is difficult to fish with bottom trawling or long lines. Then, it is likely that these areas serve as refugia from trawling and other fishing gears.

This research also helped to identify the strengths and weaknesses of the ROV as a tool to study the fish communities associated with natural habitats. Indeed, the observation of only 51 fish taxa (of which only four crypto-benthic species, such as *S*. *arenai*, *S*. *maderensis*, *Parablennius rouxi* and *Gobius* spp.) could probably mean that the ROV did not allow for a complete description of the fish assemblage, associated with these natural habitats. Similar conclusions were drawn by Andaloro *et al*. [41] and Consoli *et al*. [52] in artificial habitats (extractive platforms and shipwrecks) located in the Mediterranean Sea. According to these authors, the ROV is unable to identify crypto-benthic species, due to their small size and to their tendency to hide in holes or crevices. By contrast, according to Tessier *et al*. [62] and Andaloro *et al*. [41], the ROV is an appropriate method for censusing planktivorous fish, both from a qualitative and quantitative point of view, mostly in relation to their high abundance and low mobility.

Moreover, it is important to highlight the absence of impacts on marine habitats when ROV is chosen as tool for scientific investigations, in particular if compared with traditional invasive methodology of sampling fishing gears (e.g.: trawling). For this reasons, ROV can be used to explore sensible habitats, filming at dawn and dusk by means of highly sensitive cameras, recording information on geographic position and depth of each observed specimen and, lastly, gathering data for long periods [23].

Nowadays fundamental ecological processes that maintain bank-associated fish communities are still poorly understood. Fish diversity is only one feature of the complex bank ecosystem and several other aspects should be investigated in order to understand how oceanographic and ecological variables control the development of benthic biocenosis and the associated fish assemblages. Banks may represent important recovery areas for fish resources that could spill over towards nearby fishing grounds. The evidence put forward here, proves that banks serve as reservoirs of abundance and biodiversity and that immediate environmental conservation actions represent a priority not only for Italy but also for other countries which share the same area. The creation of a SPAMI including these banks could be a possible solution for the protection of this valuable ecosystem and for the improvement of fish stock status in the area.

## Supporting Information

S1 Dataset PRIMER matrix with densities of fish species according to “depth” and “complexity” factors. (XLSX)

S2 Dataset Fish abundances dataset used for correspondence analyses. (XLSX)

S3 Dataset Number of specimens censused at each depth. (XLSX)

S4 Dataset Whole dataset with abundances of 52 fish species. (XLSX)
